# Incorporation of humic acid into biomass derived carbon for enhanced adsorption of phenol

**DOI:** 10.1038/s41598-019-56425-8

**Published:** 2019-12-27

**Authors:** Min Song, Bing Song, Fanyue Meng, Dandan Chen, Fei Sun, Yuexing Wei

**Affiliations:** 1 Key Laboratory of Energy Thermal Conversion and Control of Ministry of Education, School of Energy and Environment, Southeast University, Nanjing, Jiangsu 210096 China; 2 School of Energy & Mechanical Engineering, Nanjing Normal University, Nanjing, Jiangsu 210023 China

**Keywords:** Pollution remediation, Natural hazards

## Abstract

In the present work, the biomass derived carbon decorated with humic acid (HC), was synthesized through impregnation method for the adsorption of phenol from water environment. Humic acids contain more oxygen-containing functional groups and hydrogen bonds, which promotes the binding between HC and phenol molecules. The results indicated that the adsorption performance of HC to phenol was better than that of commercial activated carbon. Moreover, in addition to physical absorption, the chemical reaction between carboxylic groups on the carbon surface and hydroxyl in phenol also played an important role during the process. The adsorption behavior of HC was described by equilibrium and kinetics parameters. Pseudo-second order model can describe the adsorption process well. Langmuir model was more suitable for the equilibrium adsorption data fitting, indicating that the adsorption mechanism of phenol on carbon surface tends to be monolayer adsorption. Considering practical application, UV_254_, chemical oxygen demand (COD) and ammonia from raw wastewater were selected as target contaminants and the corresponding adsorption experiments were carried out. The results displayed that HC exhibited excellent adsorption performance, especially for UV_254_, indicating that as-prepared carbon material had potential application for the control of certain organic pollutants in actual wastewater.

## Introduction

Activated carbon (AC) has received increasing attention in the past several decades due to its excellent properties such as large surface area, thermal stability, and rich surface functional groups^1,2^. With the excellent texture and surface properties, AC is a suitable material for adsorption treatment of some organic compounds in wastewater. However, the practical application of AC in wastewater treatment is limited by its high cost^3,4^. Therefore, recently, a large amount of researches focused on the development of AC derived from various carbonaceous materials such as biomass and solid wastes^5,6^. The synthesis of AC from rice husk and sawdust has drawn large attention in China due to their high carbon content and huge reserves. Mohammed *et al*.^7^ prepared activated carbon using Acacia mangium wood as raw material and the influence of chemical agents as well as activation atmosphere on the morphology of the formed carbons were investigated. Rhodamine B (Rh B) and methylene blue (MB) dyes were selected as target pollutants to estimate its adsorption activity. The results indicated that AC treated by H_3_PO_4_ was possessed of good adsorption performance for Rh B and MB. Liu *et al*. synthesized a bamboo-based AC by using microwave-induced activation with H_3_PO_4_ as activating agent^8^. The surface area of bamboo-based carbon was 1432 m^2^/g and its pore structure was developed, which was expected to be widely used as a good adsorbent. In addition, activated carbons with good physical and chemical properties were also prepared by chitosan^9^, sugarcane bagasse^10^, Jatropha hull^11^ and other biomass materials^12,13^. However, biomass derived carbon also had its inherent disadvantages. One of the most obvious defects was that there were fewer functional groups on the surface of carbon, which significantly limited its adsorption performance for the removal of organic matters in water^14,15^. Therefore, the surface characteristic of biomass derived carbons need to be improved by modification.

In recent years, humic acid (HA) has been widely used as adsorbent to remove pollutants in water^16–18^. There are two advantages for the utilization of HA. On the one hand, it could be extracted from brown coal, soil, peat and lake, so the cost of HA is low. On the other hand, HA molecule contained abundant functional groups, such as hydroxyl group, alcoholic hydroxyl group, phenolicydrazine-type carbonyl group and ketone-type carbonyl group. These groups can interact with metal ions, oxides, hydroxides, minerals, organic matters and toxic active pollutants in the environment, which can improve the hydrophilicity, adsorption and complexation of HA. Previous studies have shown that humic substances had potential application prospects for solid adsorbent modification for the remediation of contaminated water. Radwan *et al*.^19^ proposed a novel humic acid-carbon hybrid material with excellent adsorption capacity toward phenol due to its larger surface area and abundant oxygen-containing functional groups on the surface of carbon. Moreover, the novel material could be employed under a wide range of environmental conditions. Vinod *et al*.^20^ studied the adsorption performance of humic acid immobilized pillared clay (HA-PILC) for MB, crystal violet (CV), and Rh B. The results demonstrated that HA-PILC was an excellent media for cleaning treatment of organic matters in water environment.

In this study, humic acid is selected and loaded on the activated carbon from rice husk. The surface properties of activated carbon were modified and functional groups were introduced to carbon surface. Phenol in industrial wastewater is selected as target contaminant. The adsorption performance of as-prepared samples to typical organic pollutants was tested. The effect of contact time, temperature, and pH were studied. Moreover, the adsorption isotherm and kinetic were also investigated in this study, which can offer available information for optimizing treatment process of organic pollutants from wastewater.

## Results and Discussion

### Morphological properties

**Figure 1:**
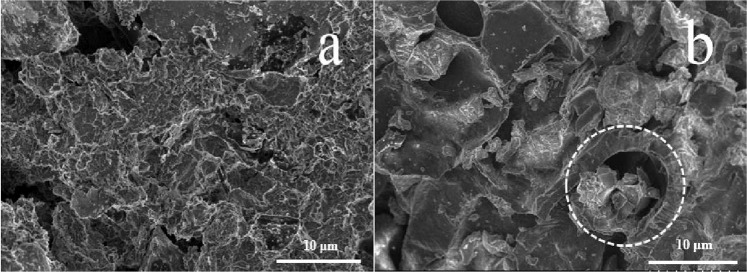
SEM photographs of (**a**) RC and (**b**) HC.

SEM photographs of rice husk-carbon (RC) and humic acid-carbon (HC) were recorded in Fig. 1. Based on Fig. 1(a), a good deal of mesopores was distributed irregularly on the RC surface, which provided a large specific surface area for the contact and adsorption of organic pollutants. The pore structure of biomass derived carbon modified by humic acid (HA) was developed, whereas partial pores were blocked (Fig. 1(b)), leading to the reduction of available surface and active sites.

The structural parameters of original and modified carbon were both listed in Table 1 and Figs. S1, S2. It can be clearly observed that RC was possessed of a high surface area of 1196.79 m^2^/g and a large pore volume of 0.3037 cm^3^/g. However, the pore structure of HA was poor, with a specific surface area of only 1.17 m^2^/g and its pore volume was too small to be detected. Although the proportion of RC in the modified sample was only 50%, but as prepared adsorbent, HC, still had considerable specific surface area of 681.47 m^2^/g. Furthermore, for HC prepared through recombination of HA and RC with a mass ratio of 1:1, it was interesting that its specific surface area did not decrease proportionately with its composition as expected, but was greater than the sum of that of them (598.98 m^2^/g). This is because ultrasonic treatment of the sample during preparation, which helps to remove impurities distributed on carbon surfaces or in channels, thus increasing the specific surface area of the sample.

**Table 1:** Structural parameters of various samples.

Samples	S_BET_ (m^2^/g)	S_mic_ (m^2^/g)	V_mic_ (cm^3^/g)	V_t_ (cm^3^/g)	D_p_ (nm)
RC	1196.79	682.49	0.3037	0.7362	2.46
HC	681.47	340.25	0.1488	0.4781	2.81
Humic acid	1.17	—	—	0.0858	2.94

Note: S_BET_: Brunauer-Emmett-Teller surface area; S_mic_: micropores surface area; V_mic_: micropores volume; V_t_: total pore volume; D_p_: average pore diameter.

### Static adsorption

**Figure 2:**
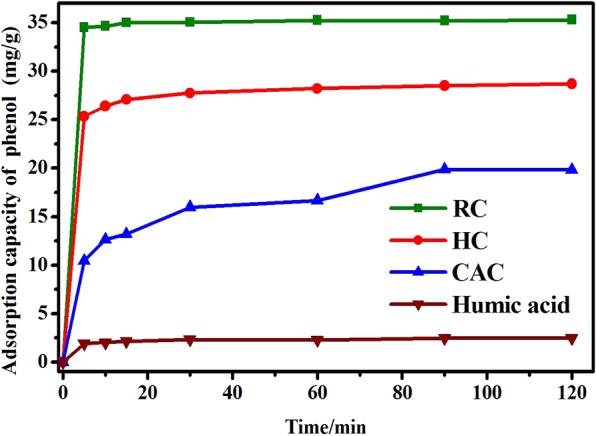
The adsorption performance of various samples for phenol with adsorption time.

Adsorption time is an important factor affecting the adsorption of phenol from solution by carbon based composite, as demonstrated in Fig. 2. The initial concentration of phenol was 40 mg/L. Because of its excellent textural properties, RC exhibited the best adsorption performance to phenol. The adsorption capacity of HC was slightly inferior than RC, but it also significantly exceeded that of commercial activated carbon (CAC) and HA. Moreover, the adsorption rate of adsorbents also varied. The adsorption amounts of RC and HC for phenol increased sharply in the first 20 min and arrived at equilibrium after 30 min. The adsorption rate of CAC was slower than that of RC and HC and it reached equilibrium after 90 min. Interestingly, the adsorption capacity of HA was rather poor, but its adsorption rate was the fastest among the four adsorbents and it reached adsorption saturation within less than 10 min. The results demonstrated that compared to CAC, HC required less residence time and presented more excellent adsorption property for organic pollutants in water environment. Additionally, for the composite adsorbent comprised of RC and HA at the rate of 1:1 in mass, its adsorption capacity was not less than half of that of RC, but reached 80.88% of that of the latter. It might be attributed to the following reasons: (i) developed pore structure; (ii) uniform distribution of HA with abundant oxygen-containing functional group on carbon surface. The hydrogen bond between oxygen in phenol molecules and hydrogen in oxygen-containing functional groups could be formed and facilitate the combination between adsorbate and adsorbent, resulting in a good adsorption capacity. Despite that adsorption capacity of HC was not extremely desirable, but it had fairly low cost compared to RC and CAC. Therefore, the as prepared carbon decorated with HA had great potential on adsorption treatment of phenol from water environment.

### Effects of pH

**Figure 3:**
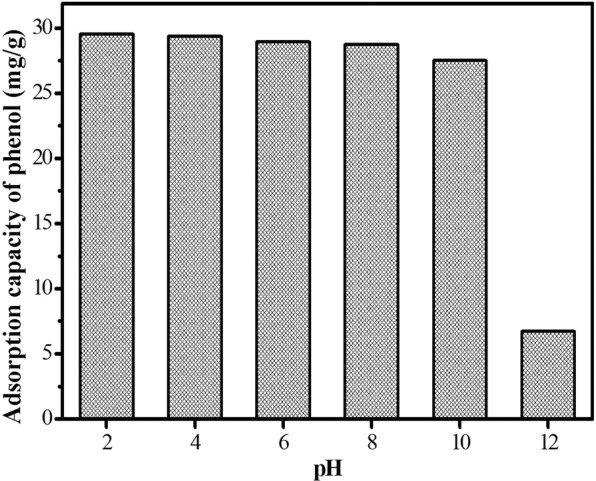
Effect of pH on the removal of phenol.

The removal of phenol versus pH was recorded in Fig. 3. The adsorption performance of HC decreased with pH. At acidic conditions, the adsorption capacity of HC was almost unchanged, and decreased rapidly when pH was greater than 10. It could be assigned to the variation of surface charge on the adsorbent and the solubleness of adsorbate at different solution pH^21,22^. As a weak acid^23^, phenol was dissociated when pH > pK_a_ and was transformed into phenolate-phenolate anions at alkaline pH due to the ionization of phenol molecules. Therefore, the reason for the decrease of adsorption amount might be assigned to electrostatic repulsions between negative surface charges caused by OH^-^ at high pH and the phenolate anions^24^. While in acidic environment, the adsorption capacity remained almost unchanged because phenol was undissociated and dispersion interaction predominated.

### Adsorption kinetics

To better illustrate the adsorption behavior of phenol on HC surface, pseudo-first-order kinetics model and pseudo-second-order kinetics model were used to fit the data. The former model assumed that adsorbate molecules were proportionally bound to the active sites, whereas in the latter model adsorbate molecule was adsorbed onto two active sites^25^. According to Trivedi^26^ and Ho (Mckay)^27^, the equations of the two kinetic models were given as:

1 $$qt=qe(1-\exp (\,-\,k1t))$$

2 $$\frac{t}{qt}=\frac{1}{k2{q}_{e}\cdot {q}_{e}}+\frac{t}{qe}$$

where *q*_e_ (mg/g) is adsorption capacity of adsorbent at equilibrium, *k*_1_ (min^−1^) and *k*_2_ (mg/g • min) are rate constants, *t* (min) is adsorption time.

The kinetics parameters calculated through the equations were recorded in Table 2. The correlation coefficients (R^2^) and q_e_ were utilized to confirm the suitability of two models. The R^2^ value of pseudo first order kinetics model was fairly low, whereas that of pseudo second order model was larger than 0.99. The calculated equilibrium adsorption capacity was also accorded with actual adsorption capacity of as prepared adsorbent according to pseudo-second-order model, which demonstrates that this model could better depict the adsorption behavior.

**Table 2:** Kinetics parameters for phenol adsorption.

Absorbate	Adsorbent	Pseudo-first order			Pseudo-second-order		
		q_e_(mg/g)	k_1_(g(mg/min)^−1^	R^2^	q_e_(mg/g)	k_2_(g(mg/min)^−1^	R^2^
phenol	HC	72.94	0.546	0.79	17.28	8.74	0.996

### Adsorption isotherm

**Figure 4:**
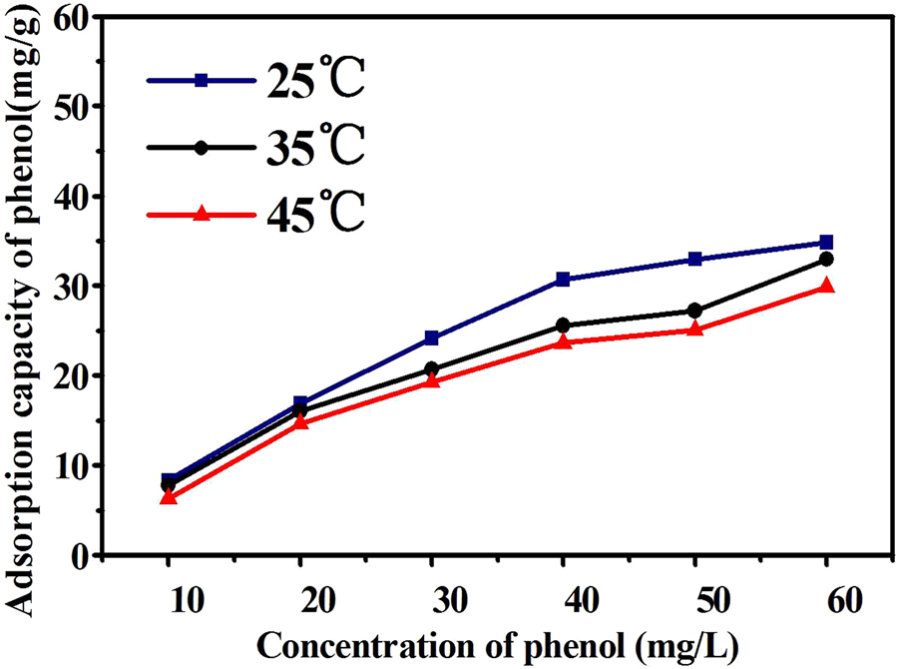
Adsorption isotherms of phenol on HC.

The adsorption was performed at various temperatures with various initial concentrations of organic compounds from 10 mg/L to 60 mg/L. The impact of temperature on migration and adsorption behavior of phenol on HC surface were demonstrated in Fig. 4. On the one hand, the absorption capacity of adsorbent for phenol decreased to varying degrees with the increase of temperature and initial concentration. It could be inferred that the adsorption reaction was an exothermic process, the interaction between adsorbent and organic compound (i.e. electrostatic force and hydrogen bond) decreased with increasing temperature, resulting in a decrease in adsorption capacity. On the other hand, the average pore size of HC was 2.81 nm (see Table 1), which was bigger than the kinetic diameter of phenol. The larger pore was conducive to accelerate the adsorption rate and aggravate the intra-particle diffusion. Additionally, the growth rate of adsorption capacity decreased gradually with increasing initial concentration. This is mainly because the diffusion resistance of the organic compound would increase in gradually the narrow pores with the increasing concentration of pollutant.

**Figure 5:**
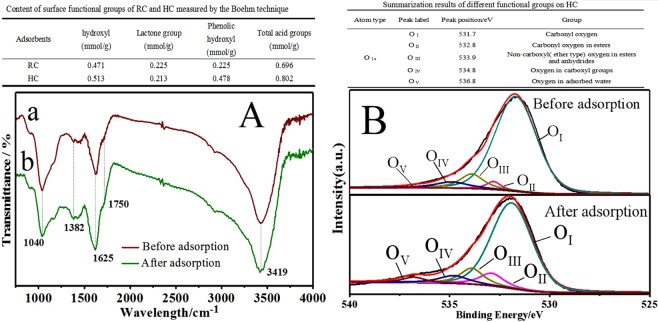
Typical FTIR spectra (**A**) and XPS (**B**) of the starting and tested adsorbents.

Furthermore, the adsorption of HC to phenol decreased with the increasing temperature, which demonstrates that there was chemical interaction between adsorbate and oxygen-containing functional groups^28^. Thus, to clarify the interaction and figure out the binding mechanism between the adsorbent and phenol, FTIR, Boehm titration, and XPS were applied. Based on Fig. 5(A), the peaks at 1040 and 1625 cm^−1^ were respectively assigned to C-O and C=C stretching. The band at 3450 cm^−1^ was referred to OH stretching of H-bonded water and it was unchanged before and after adsorption. Additionally, the relative strength of the peak assigned to carboxylic acid groups (1750 cm^−1^) decreased after adsorption, whereas the relative strength of ester groups (1382 cm^−1^) increased. This is also consistent with the data in Table S1. It could be inferred that, in addition to physical absorption, the chemical reaction between carboxylic groups on the carbon surface and hydroxyl in phenol also played a role in the removal process of phenol from aqueous solution by the modified carbon. Furthermore, based on the interpolation table of Fig. 5(A), more hydroxyl groups, lactone groups and phenol hydroxyl groups on the HC surface were observed compared to that on the RC surface. These groups were derived from HA which contains rich acidic functional groups. The increase of functional groups might be beneficial to enhance the adsorption performance of HC to phenol. The XPS spectra of O 1 s of samples before and after adsorption were shown in Fig. 5(B). The absorption peak appearing in the range of 540 to 528 eV was corresponded to O 1 s. It could be clearly observed that O 1 s peak was deconvoluted into five Gaussian symmetric curves at 531.7, 532.8, 533.9, 534.8, and 536.8 eV. The corresponding details of these peaks were listed in the interpolation table of Fig. 5(B). The peak area corresponding to ester group was significantly increased, demonstrating the increase of ester groups in HC after adsorption. The result indicated that new ester groups were formed during the adsorption process which was in good agreement with the FTIR data.

In order to predict the modelling procedures, the data derived from the adsorption experiment of HC to phenol were fitted by the Langmuir and Freundlich isotherm models. According to Langmuir^29^ and Hameed^30^, the linear formulas of Langmuir and Freundlich model were described as:

3 $$\frac{1}{qe}=\frac{1}{{k}_{1}qm}\ast \frac{1}{Ce}+\frac{1}{qm}$$

4 $$\mathrm{ln}\,qe=\,\mathrm{ln}\,{k}_{2}+\frac{1}{n}\,\mathrm{ln}\,Ce$$

where *q*_e_ (mg/g) is adsorption capacity of adsorbent at equilibrium, *q*_m_ is theoretical monolayer adsorption capacity, *C*_e_ is equilibrium concentration of adsorbing, *k*_1_, *k*_2_ and 1/*n* are adsorption constants.

The parameters corresponding to the Langmuir and Freundlich model were listed in Table 3. The results showed that the adsorption performance of phenol was better fitted by the first model. It could be deduced that the adsorption behavior of the organic pollutant on HC surface was inclined to follow monolayer adsorption mechanism. Furthermore, the values of 1/*n* were arranged from 0.49 to 0.71, which implied favorable adsorption for phenol on the carbon surface.

**Table 3:** Isotherm parameters for HC.

Absorbate	Adsorbent	Langmuir model			Freundlich model		
		q_m_(mg/g)	K_L_ (L/mg)	R^2^	K_F_ (L/g)	1/n	R^2^
Phenol	HC	58.89	0.105	0.9848	8.649	0.49	0.9167

Table 4 enumerated the maximum adsorption capacities of different carbon based adsorbents to phenol, which were derived from corresponding literatures. It can be concluded that the carbon based adsorbent investigated in this work had excellent adsorption performance to the organic pollutant in aqueous solution.

**Table 4:** Comparison of organic pollutant removal by various adsorbents.

Adsorbents	Adsorbate	Isotherm model						References
		Langmuir model			Freundlich model			
		q_m_, mg/g	K_L_, L/mg	R^2^	n	K_F_	R^2^	
1.2SEP/C	Phenol	5.26	0.0265	0.993	1.54	0.21	0.950	^31^
Bentonite (surfactant)	Phenol	22.88	0.011	0.996	31.8	3.4	0.958	^32^
Granular activated carbon	Phenol	112.36	0.013	0.996	1.28	3.03	0.981	^33^
Amine-modified activated carbon	Phenol	18.12	0.000059	0.999	3.11	0.39	0.983	^34^
MWCNTs	Phenol	32.25	3.17	0.990	1.76	1.11	0.896	^35^
HC	Phenol	58.89	0.1050	0.984	2.02	8.64	0.917	This work

### Adsorption of actual wastewater

**Figure 6:**
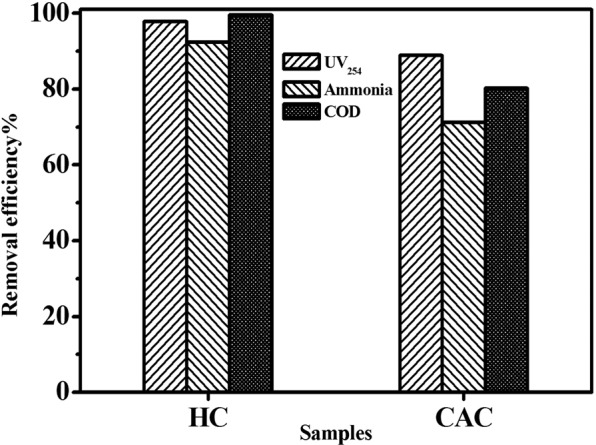
The removal rate of UV254, ammonia, and COD in practical wastewater by different adsorbents.

In order to further investigate practical application on the removal of organic compounds, HC and commercial activated carbon (CAC) were selected as adsorbents for the adsorption experiments to certain organic pollutants from actual wastewater. Concentrations of the pollutants could reflect the content of macromolecular organic matters and the aromatic compounds containing C=C double bond or C=O double bond present in water. Therefore, ammonia, COD, and UV_254_ were chosen as target contaminants in the work to evaluate the ability of adsorbents to remove organic pollutants in wastewater. The adsorption experiments were performed based on the same experiment procedures (as described in Section 2.5) and the result was shown in Fig. 6. It was clearly observed that as prepared sample displayed favourable removal efficiency for ammonia, COD, and UV_254_. Moreover, its performance was apparently superior to commercial activated carbon. The COD concentration was reduced from 212 mg/g of the original wastewater to less than 5 mg/L of the treated wastewater which was far below the sewage discharge standard value (GB8978–1996) (Table S2), and the removal rate reached 99.52%. The above results indicated that the as prepared carbon based materials have potential application prospects in practical wastewater treatment.

In conclusion, this study exhibited the potential of the rice husk-based carbon coated with humic acid for phenol removal from aqueous solution. HC was possessed of high surface area and abundant oxygen-containing functional groups, and it exhibited excellent adsorption performance for phenol. The optimum removal condition of HC was attained at pH = 6, 25 °C and 30 min contact time. The experimental data derived from the adsorption process of the carbon based adsorbent for phenol was best fitted by the Langmuir adsorption isotherm model. The kinetic results demonstrated that the process was better described through pseudo second-order model. Based on the adsorption results of COD, UV_254_, and ammonia from raw water, it could be concluded that as prepared carbon based materials can be employed as highly efficient adsorbents used for the disposal of organic pollutants in practical wastewater.

## Methods

Rice husk was obtained from Shandong, China. Hydrochloric acid (HCl), potassium hydroxide (KOH), phenol was all provided by Sigma-Aldrich. The elemental analysis and industrial analysis for rice husk were shown in Table 5. It can be inferred that the raw material is in rich of carbon element and is suitable for the synthesis of activated carbon. Phenol used in this study was analytically pure grade and its main properties were shown in Table 6^36,37^.

**Table 5:** Elemental analysis and industrial analysis of rice husk.

Material	Elemental analysis/%				Industrial analysis /%			
	C_ad_	H_ad_	N_ad_	O_ad_	V_ad_	FC_ad_	A_d_	M_ad_
Rice husk	50.07	6.07	0.63	43.23	67.28	11.61	12.92	8.18

Note: Ad: air dried basis; d: dried basis

**Table 6:** Selected physicochemical properties of the adsorbate ^a^.

Adsorbate	MW	C_s_	⌊_max_	pK_a_	^□*^	〈_m_	®_m_	d_m_	〉	MP
phenol	94.11	80190	269	9.9	0.72	0.61	0.33	0.69	1.071	40.9

Note: ^a^MW: molecular weight (g/mol); C_s_: water solubility (mg/L) (at 298 K); λ_max_: maximum adsorption wavelength (nm); pK_a_: dissociated constant (at 298 K); π*: polarity/polarizability parameter; α_m_: hydrogen-bonding donor parameter; β_m_: hydrogen-bonding acceptor parameter; d_m_: molecular dynamics diameter (nm); ρ:density (g/cm^3^); MP: melting points (°C).

### Preparation of biomass derived carbon

After pretreatment, a certain amount of rice husk was mixed with KOH solution at KOH/rice husk weight ratio of 3:1 in a glass beaker. The mixture was stirred at 40 °C for 8 h, and was then dried in the oven at 105 °C for 24 h. The activated sample were transferred to a quartz boat and placed in a high temperature horizontal tube furnace. Under the N_2_ flow condition, the temperature was set at 800 °C and the samples were held at this condition for 60 min. Finally, the product was washed with 0.05 mol/L hydrochloric solution until the filtrate was neutral and was then dried in the air dry oven at 105 °C for 24 h. The obtained final product was named as rice husk-carbon (labeled as RC).

### Preparation of carbon based composite adsorbents

Humic acid was mixed with RC at weight ratio of 1:1. A small amount of distilled water was put into the mixture and the pH value of solution was regulated to 2 with hydrochloric solution (1 mol/L). The solution was slowly stirred by a glass rod for 15 min, ultrasonicated for 30 min, then washed, filtered and dried. The obtained composite material, humic acid-carbon, was designated as HC.

### Characterization of carbon based composite adsorbents

The porous texture of synthesized adsorbents was characterized through Surface Area and porosimetry Analyzer (V-Sorb 2800 P). The species and quantity of functional groups on carbon surface were investigated by Boehm titration method^15,38^. The microstructure of adsorbents was imaged using a scanning electron microscopy (SEM) (Hitachi, S-3000N), operating at a 20 kV accelerating voltage. The functional groups on samples surface were also characterized by a Fourier transform infrared spectra (Bruker Tensor 27, Germany) in the scanning region of 500–4000 cm^−1^. The nature of chemical binding of samples was identified by XPS (VG Scienta, USA).

### Batch adsorption experiments

In the experiment, phenol was selected as the targeted contaminant. 100 mL phenol (40 mg/L) and as prepared adsorbents (0.1 g) were transferred to glass bottles. The glass bottles were mechanically shaken (150 rpm) at room temperature in a constant temperature oscillator (KYC-1102C). The concentration of phenol after filtering was then measured. The formula for equilibrium adsorption capacity was defined as follows:

5 $$qe=\frac{V0(C0-Ce)}{m}$$

where, *q*_e_ is equilibrium absorption capacity of samples (mg/g); *V*_0_ represents solution volume (ml); *C*_0_ and *C*_e_ are the concentrations of phenol at initial condition and at adsorption equilibrium condition (mg/L), respectively; *m* is the mass of adsorbent (g). For comparison, commercial activated carbons (CAC), humic acid, and RC were all used in adsorption experiments.

## Supplementary information

Supplementary Information

## Data Availability

All data generated or analysed during this study are included in this article.
