# Supplementary material for: Incorporation of humic acid into biomass derived carbon for enhanced adsorption of phenol
Source: Sci Rep. 2019 Dec 27;9:19931. doi: 10.1038/s41598-019-56425-8 (PMC6934675; doi:10.1038/s41598-019-56425-8)
Supplement: Supplementary file 1 — Supplementary Information [file 41598_2019_56425_MOESM1_ESM.doc]

**Supplementary material**

**Incorporation of humic acid into biomass derived carbon for enhanced adsorption of phenol**

Min Song^a,*^, Bing Song^a^, Fanyue Meng^a^, Dandan Chen^a,b^, Fei Sun^a^, Yuexing Wei^a^

*^a^ Ministry of Education of Key Laboratory of Energy Thermal Conversion and Control, School of Energy and Environment, Southeast University, Nanjing, Jiangsu 210096, China;*

*^b^ School of Energy & Mechanical Engineering, Nanjing Normal University, Nanjing, Jiangsu 210023, China*

* Corresponding author: Min Song

Tel: +86-13770606581; Fax: +86-025-83790986

E-mail address: [minsong@seu.edu.cn](mailto:minsong@seu.edu.cn)

**Supplementary Captions**:

**Fig. S1** N_2_ adsorption and desorption isotherm (a) and pore size distribution curve (b) of RC at 77K

**Fig. S2** N_2_ adsorption and desorption isotherm (c) and pore size distribution curve (d) of HC at 77K.

**Table S1** Content of surface functional groups of HC (before and after) measured by the Boehm technique

| Absorbents | [Carboxyl](../../../../D:/Dict/7.2.0.0615/resultui/dict/%3Fkeyword=carboxyl)  /(mmol/g) | Lactone group /(mmol/g) | Phenolic hydroxyl  /(mmol/g) | Total acid groups /(mmol/g) |
| --- | --- | --- | --- | --- |
| HC (before) | 0.513 | 0.213 | 0.478 | 1.204 |
| HC (after) | 0.472 | 0.301 | 0.468 | 1.241 |

**Table S2** The water quality index of raw water and sewage discharge standard in GB8978-1996

|  | pH | UV_254_ | Ammonia  (mg/L) | Salinity  （ppt） | Electric conductivity  (us/m) | COD(mg/L) |
| --- | --- | --- | --- | --- | --- | --- |
| Raw water | 6.989 | 0.225 | 10.383 | 0.10 | 378 | 212 |
| Standard | 6-9 | - | <15 | - | - | <100 |
